# Supplementary material for: Eribulin and Paclitaxel Differentially Alter Extracellular Vesicles and Their Cargo from Triple-Negative Breast Cancer Cells
Source: Cancers (Basel). 2021 Jun 3;13(11):2783. doi: 10.3390/cancers13112783 (PMC8199867; doi:10.3390/cancers13112783)
Supplement: Supplementary file 1 [file cancers-13-02783-s001.zip › cancers-1219707-supplementary.pdf]

SUPPLEMENTARY MATERIALS

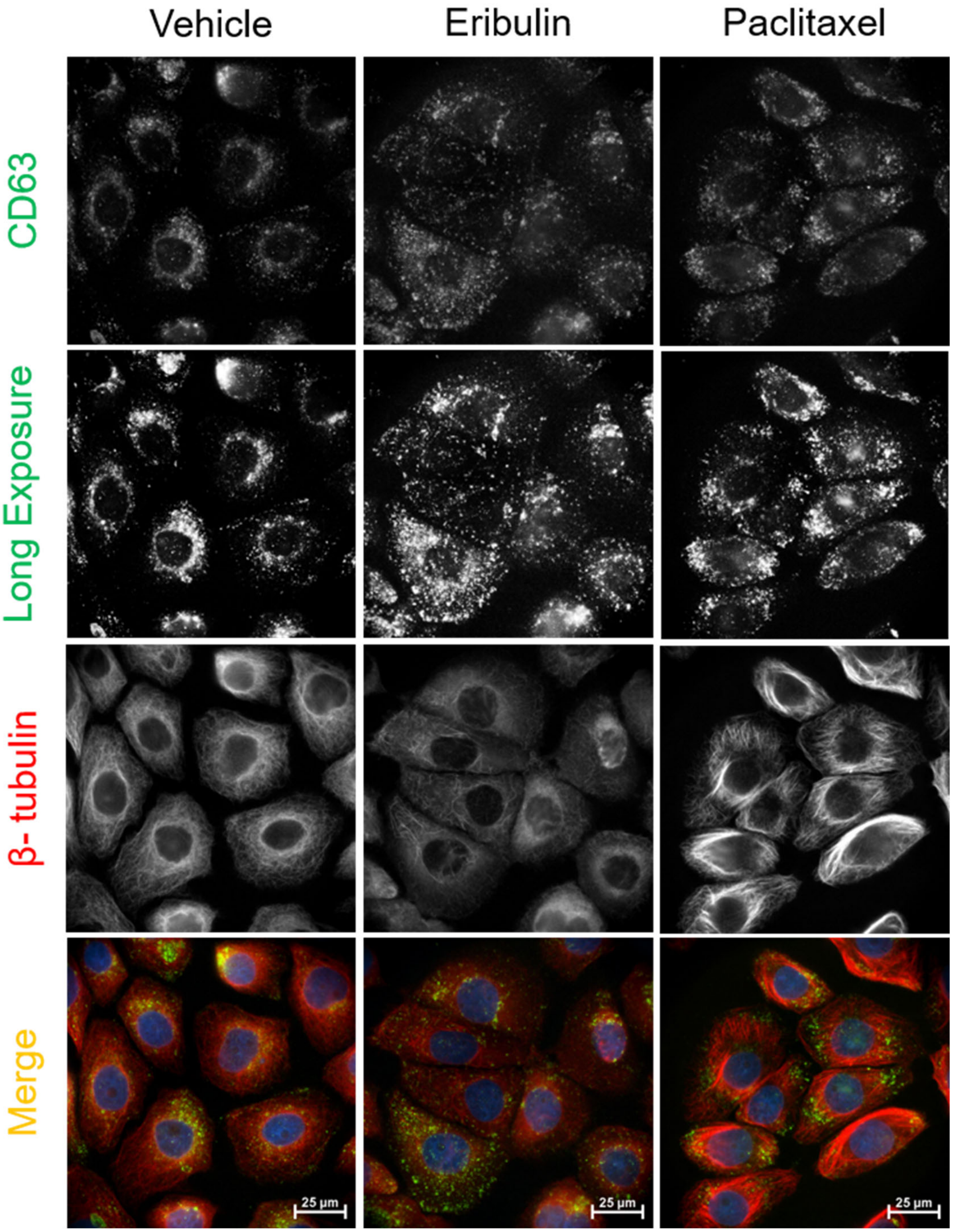

Figure S1. Effects of 2-h eribulin or paclitaxel treatment on microtubule structures and CD63 localization. HCC1937 cells were treated with vehicle, 50 nM eribulin or 500 nM paclitaxel, for 2 h and then co-immunostained for CD63 (green) and β-tubulin (red). Immunofluorescence images are representative of two experiments. Scale bar = 25 μm.

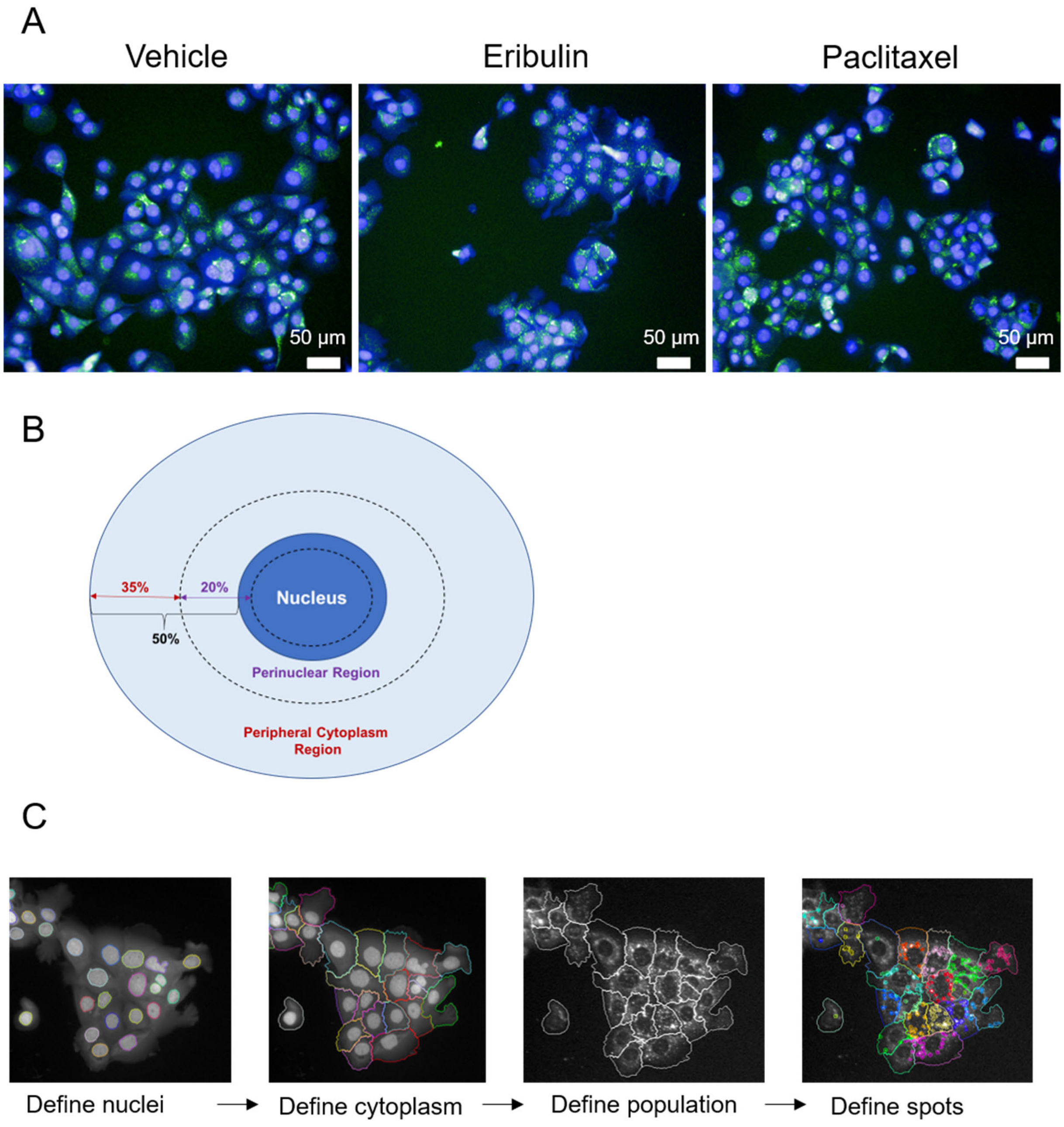

Figure S2. High content imaging analysis in HCC1937 cells**.** (**A**) Representative images, corresponding to main figure 1B and C, of cells treated with vehicle, 50 nM eribulin or 500 nM paclitaxel for 4 h and immunostained for CD63 (green) as well as cell mask blue and DAPI to identify the cytoplasm and nucleus, respectively. Scale bar = 50 µm. (**B**) Diagram illustrating the definition of cell regions used for the analysis shown in figure 1B and described in the methods section. (**C**) Diagram illustrating how DAPI and cell mask blue were used to define the population of cells for the analysis shown in figure 1C, and the selection of spots of CD63 using Method C in the Columbus™ software as described in the methods section.

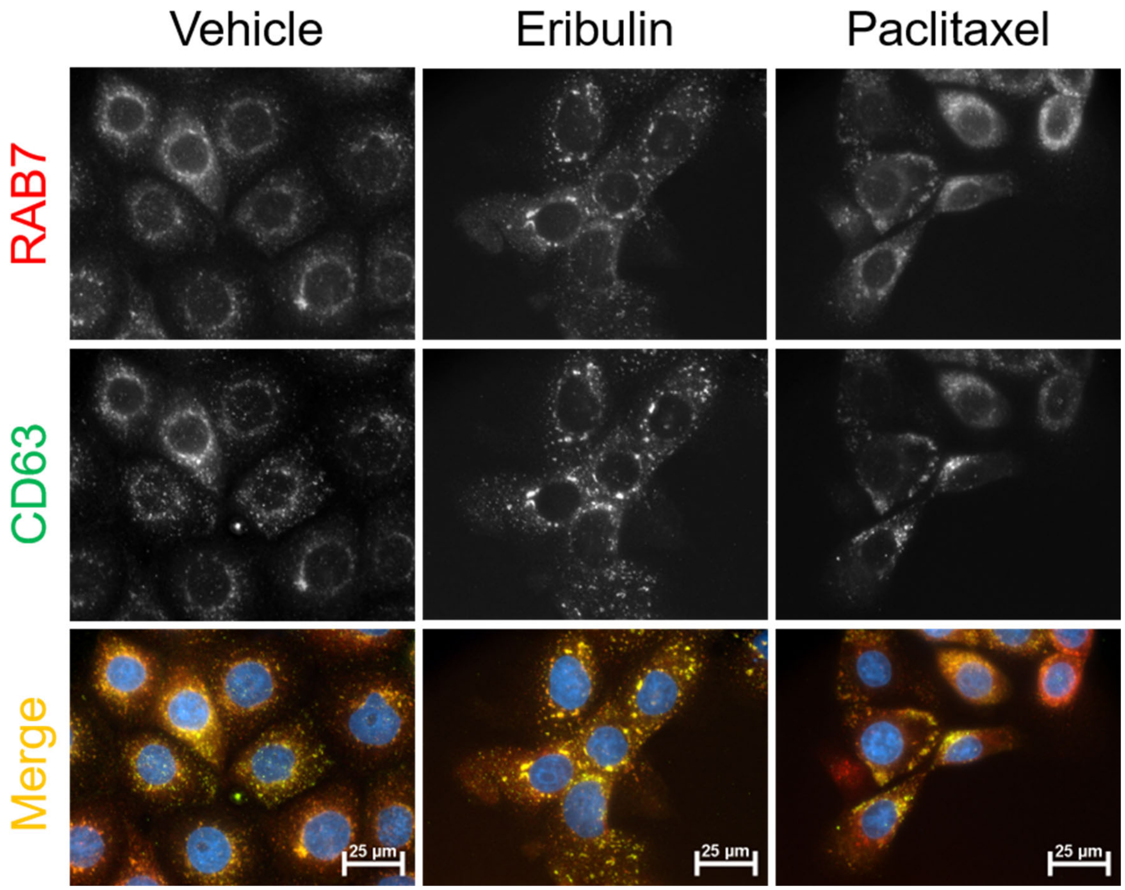

Figure S3. Effects of eribulin or paclitaxel treatment on RAB7 localization. HCC1937 cells were treated with vehicle, 50 nM eribulin or 500 nM paclitaxel for 4 h to cause maximal microtubule disruption and then CD63 (green) and RAB7 (red) were visualized by immunostaining. Pictures are representative of two experiments. Scale bar = 25 µm.

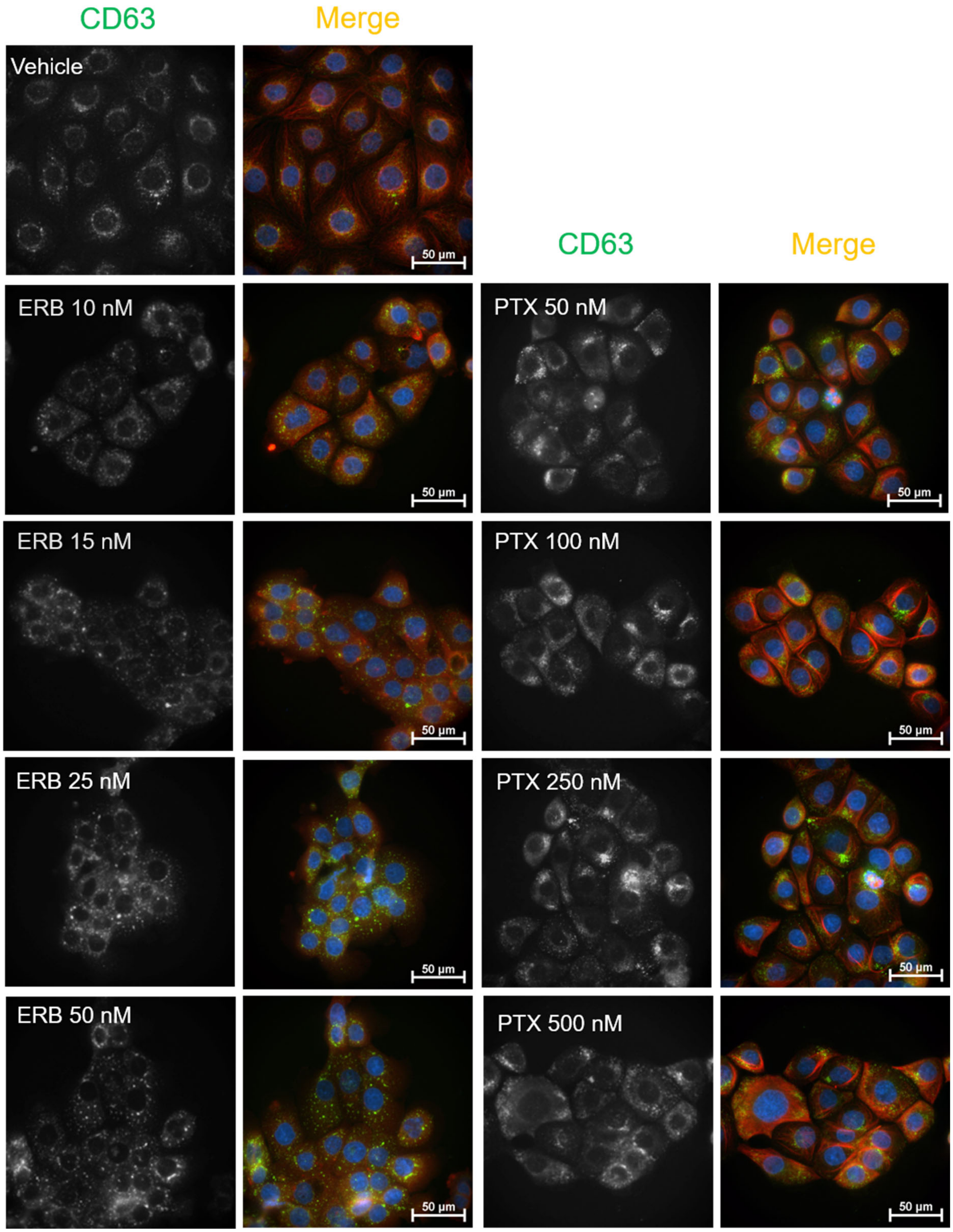

Figure S4. Concentration-dependent effects of eribulin and paclitaxel on CD63 localization. HCC1937 cells were treated with indicated concentrations of eribulin or paclitaxel for 8 h in serum-free media followed by immunostaining for CD63 (green) and β-tubulin (red). Scale bar = 50 μm.

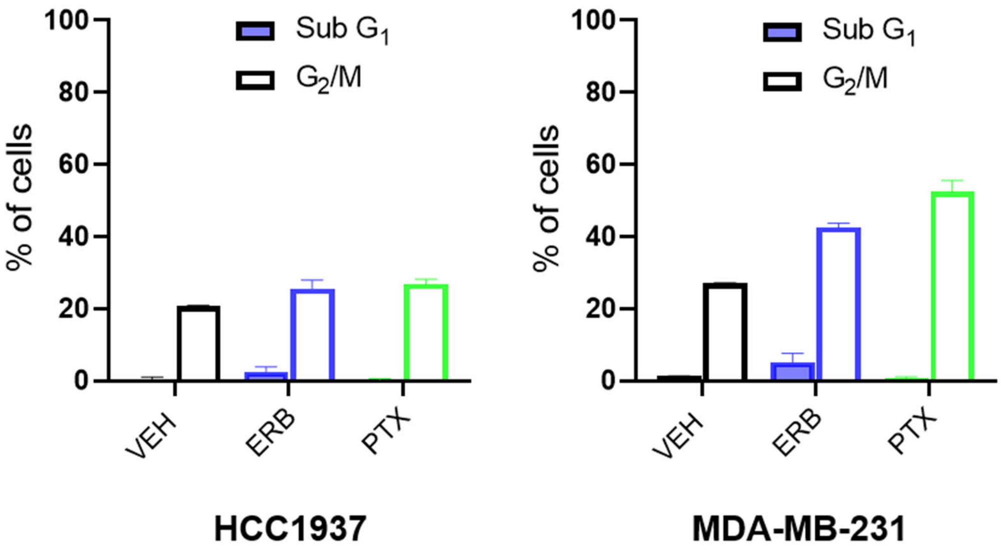

Figure S5. Effects of eribulin and paclitaxel on the percentage of cells in sub G_1 or G_2M. HCC1937 and MDA-MB-231 cells were treated with vehicle, 25 nM eribulin or 50 nM paclitaxel for 8 h in serum-free media followed by propidium iodide staining and cell cycle analysis by flow cytometry. $n = 2$

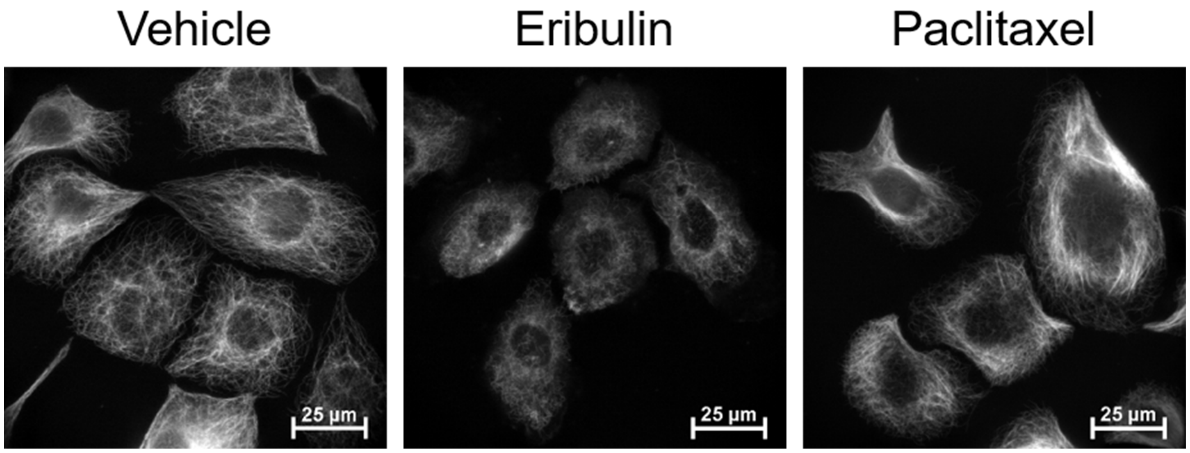

Figure S6. Microtubule disruption by eribulin or paclitaxel after 2-h treatment. HCC1937 cells were treated for 2 h with vehicle, 25 nM eribulin or 50 nM paclitaxel in serum-free media, followed by immunostaining for β-tubulin. Images are representative of two independent experiments. Scale bar = 25 µm.

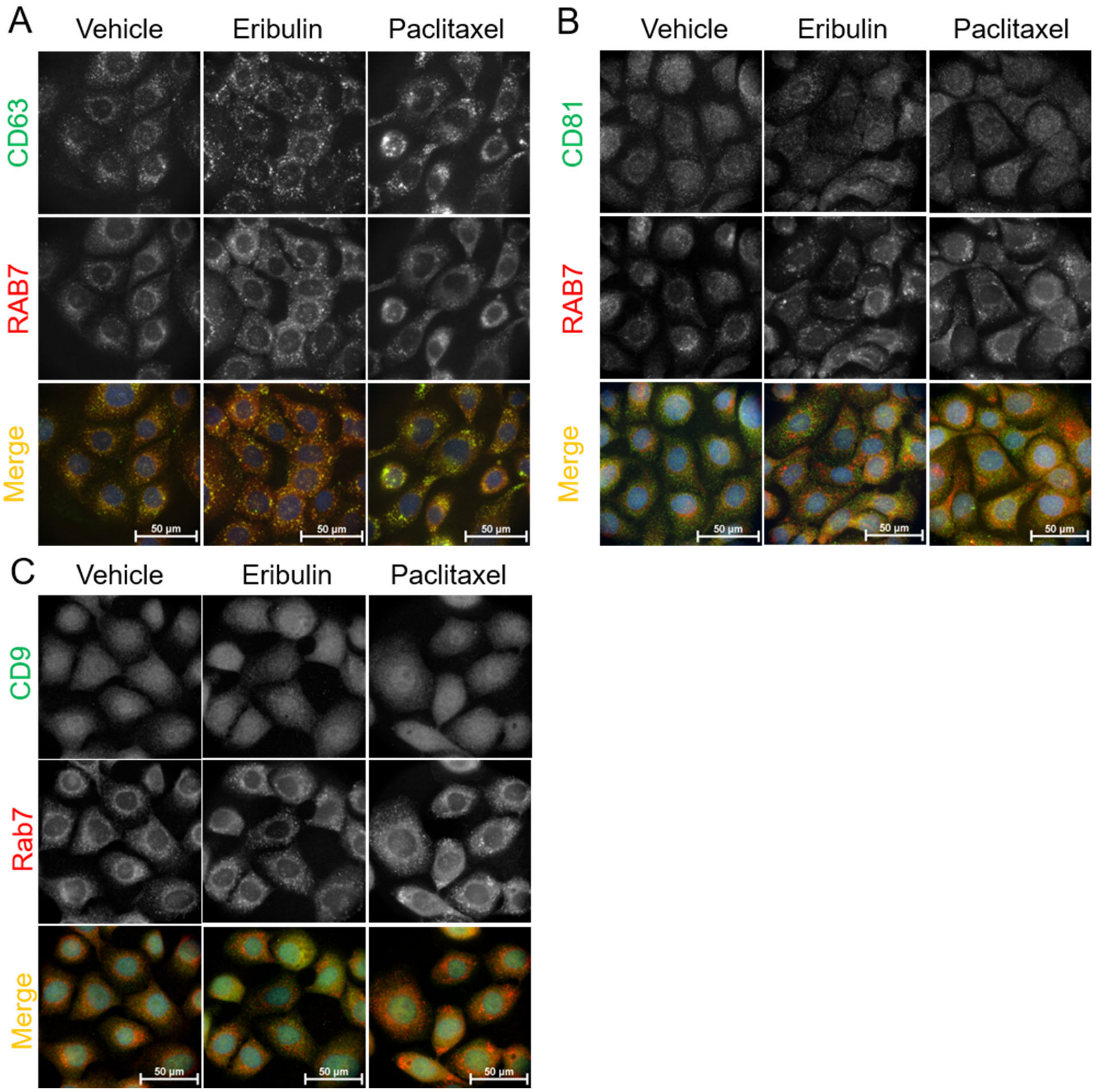

Figure S7. Effects of eribulin or paclitaxel treatment on the localization of tetraspanins. HCC1937 cells were treated with 25 nM eribulin or 50 nM paclitaxel for 4 h in serum-free media and then co-immunostained for RAB7 (red) plus CD63 (**A**), CD81 (**B**), or CD9 (**C**) (green). Pictures are representative of two independent experiments. Scale bar = 50 µm.

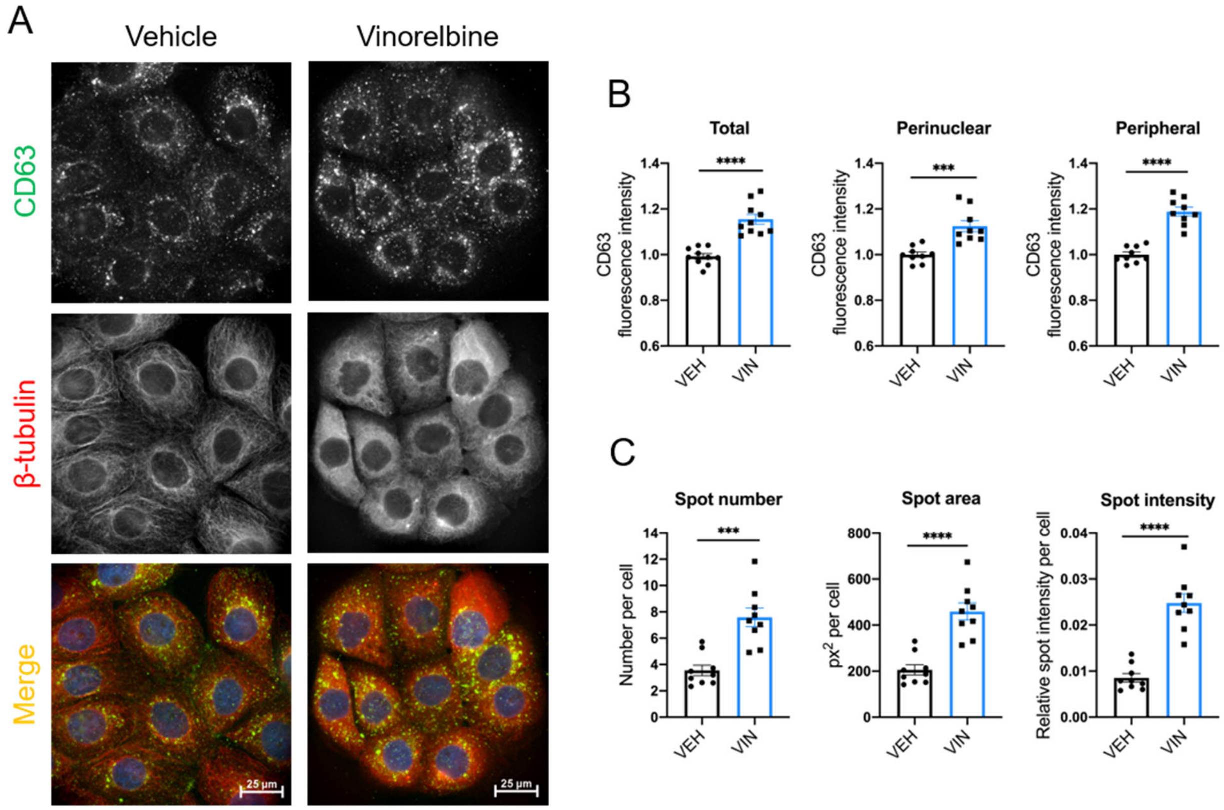

Figure S8. Effects of vinorelbine on CD63 localization in HCC1937 cells. HCC1937 cells were treated with vehicle or 50 nM vinorelbine for 4 h to cause maximal microtubule disruption and then co-immunostained for CD63 (green) and β-tubulin (red). (**A**) Representative immunofluorescence images. Scale bar = 25 μm. (**B**) Quantification of CD63 regional intensity and (**C**) CD63 spots analysis using Operetta™ high-content imaging. Mean cellular fluorescence for an average of 577 cells (range: 191–1399) was calculated for n = 9 wells and represents three independent experiments. Significance determined by unpaired t- test. *** $p < 0.001$, **** $p < 0.0001$

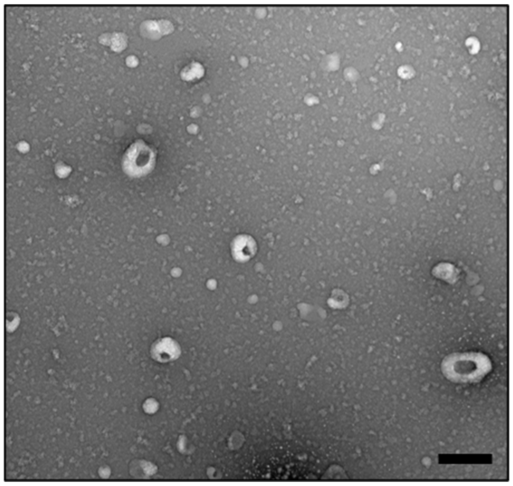

Figure S9. Electron micrograph of sEVs from vinorelbine treated MDA-MB-231 cells. Cells were treated for 8 h with 25 nM vinorelbine and sEVs were collected from the conditioned media and evaluated by transmitting electron microscopy. Scale bar = 200 nm.

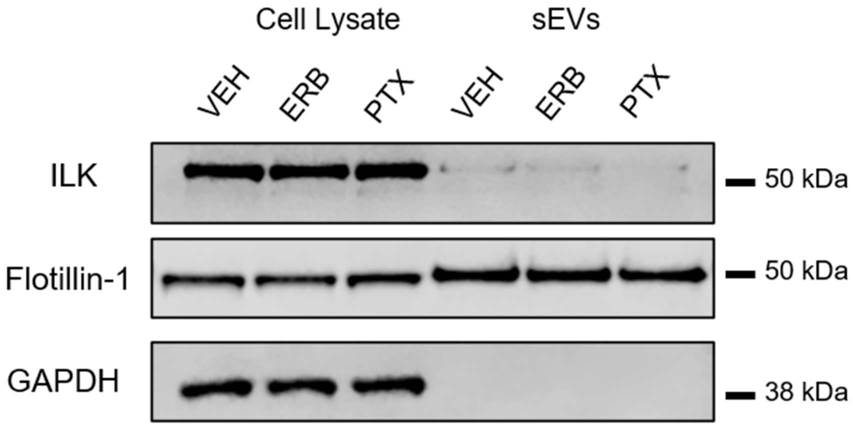

Figure S10. Evaluating ILK in MDA-MB-231 cells and sEVs. MDA-MB-231 cells were treated for 8 h with vehicle, 25 nM eribulin or 50 nM paclitaxel and sEVs were isolated from the conditioned media. Representative immunoblots for indicated proteins in 10 µg of cell lysates and equal volumes of sEVs representing 42% (ILK, flotillin-1) or 7.5% (GAPDH) of the yield from 1.2×10^8 cells.

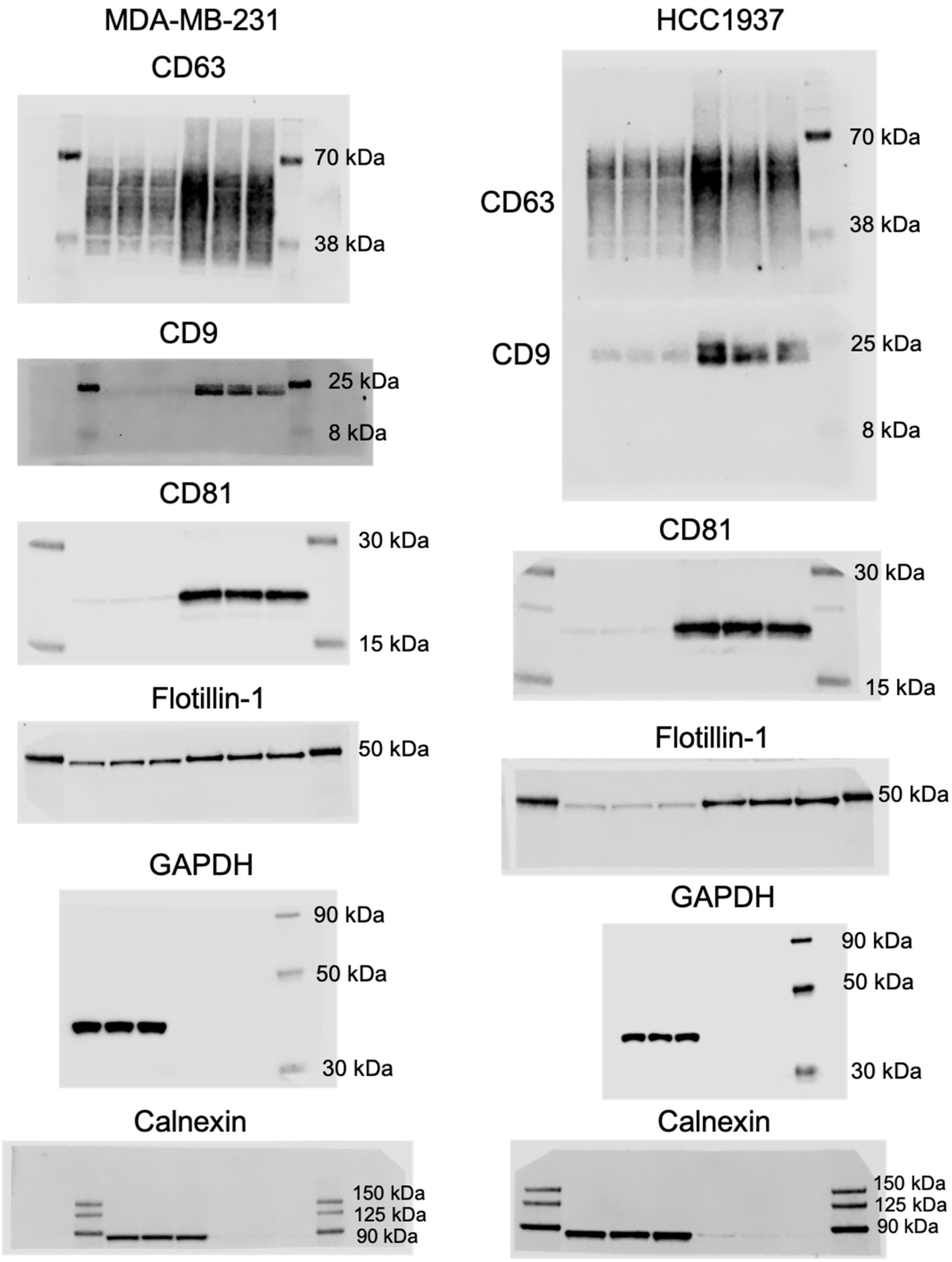

Figure S11. Uncropped Western blots corresponding to Figure 3.

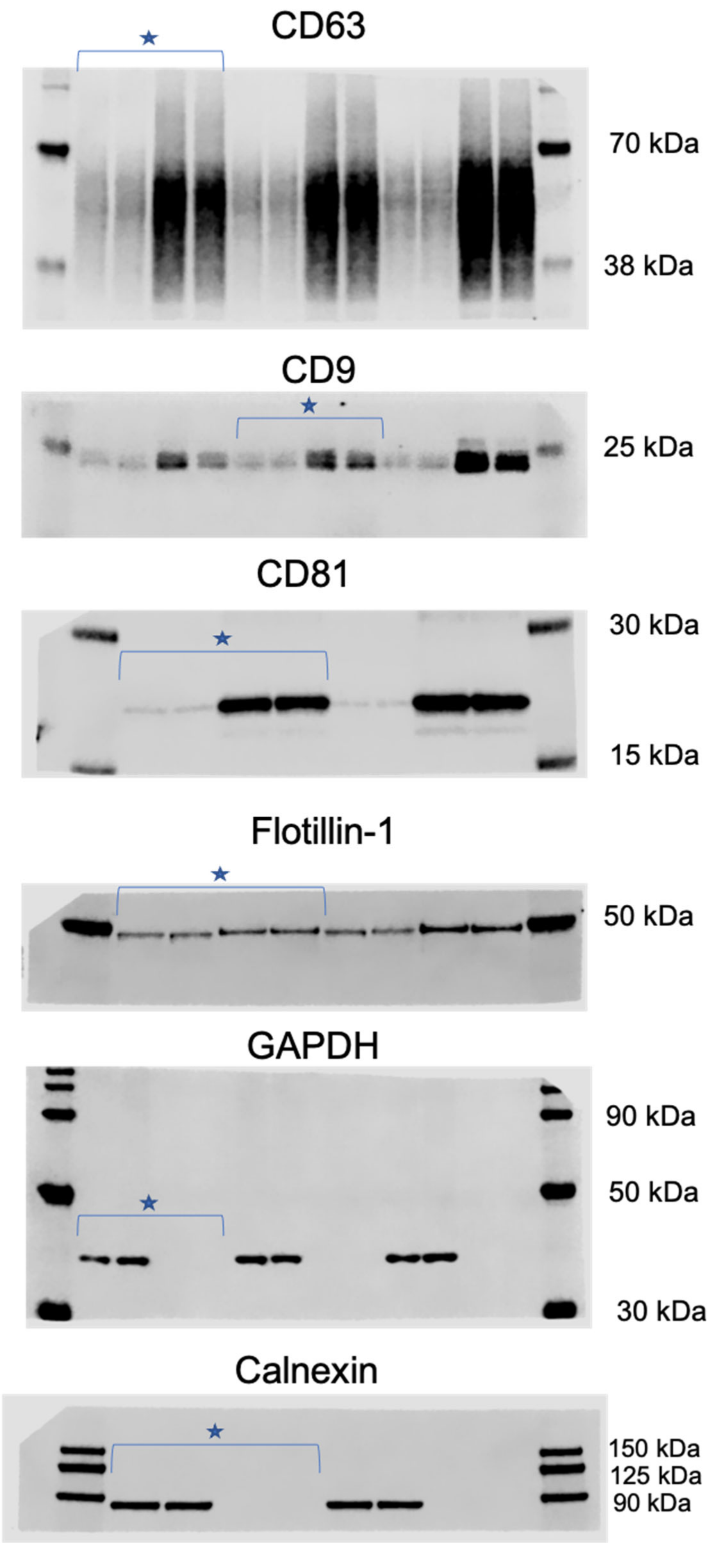

Figure S12. Uncropped Western blots corresponding to Figure 5. Sections of the blot designated with star symbol correspond to the cropped blot areas shown in the figure.

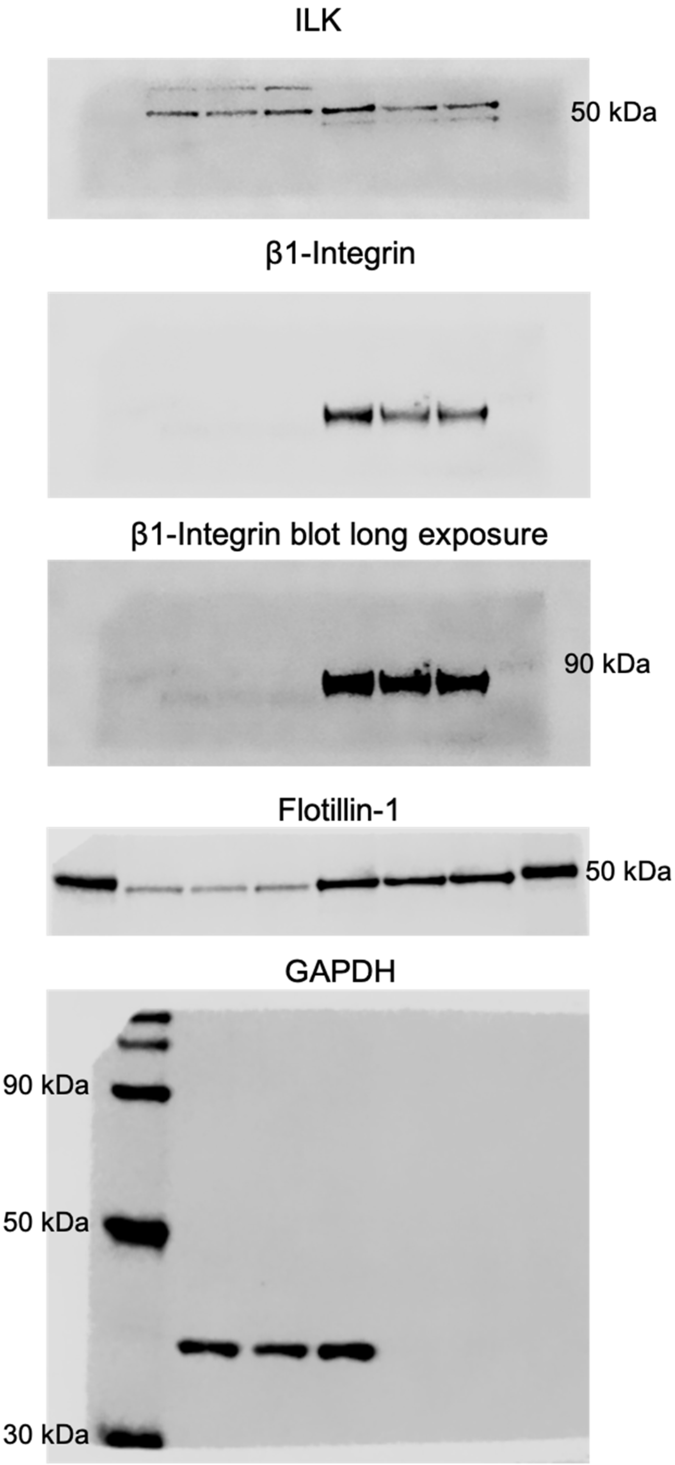

Figure S13. Uncropped Western blots corresponding to Figure 6.

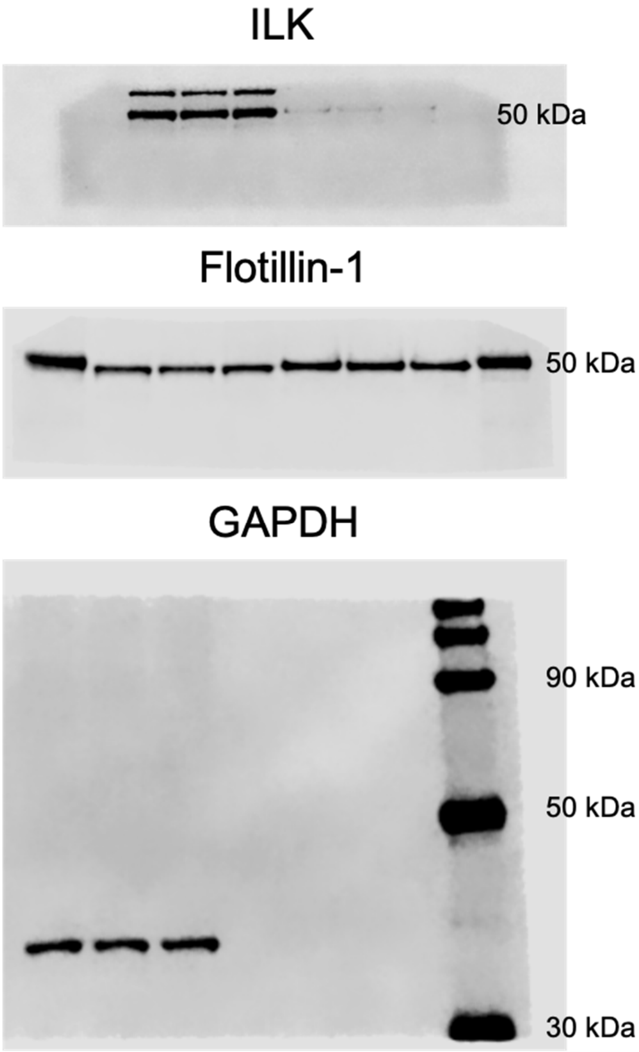

Figure S14. Uncropped Western blots corresponding to Figure S10.

Table S1. Antibody vendor and application information.

Target	Product number and Vendor	Host	Clone	Application-Dilutions
CD63	10628D Invitrogen	Mouse	TS63	WB-1:1000<br>IF- 1:400
CD9	13174s Lot:3 Cell Signaling<br>NBP2-22187, Novus Biologicals	Rabbit<br>Mouse	D801A<br>5G6	WB-1:1000<br>IF- 1:1000
CD81	Sc-166029 Santa Cruz	Mouse	B-11	WB-1:1000<br>IF- 1:100
GAPDH	5174s Lot:6 Cell Signaling	Rabbit	D16H11	WB-1:1000
Flotillin-1	18634 Cell Signaling	Rabbit	D2V7	WB-1:1000
Calnexin	2679s Cell Signaling	Rabbit	C5C9	WB-1:1000
ILK	3862 Cell Signaling	Rabbit		WB-1:1000
β-1 Integrin	4706 Cell Signaling	Rabbit		WB-1:1000
β-Tubulin	ab6046 Abcam<br>T4026 Sigma	Rabbit<br>Mouse		IF- 1:1000<br>IF- 1:1000 (Figure. S5)
RAB7	9367 Cell Signaling	Rabbit	D95F2	IF- 1:200
Anti-Mouse IgG-FITC	F3008 Sigma	Sheep		IF- 1:1000
Texas Red®-X goat anti-rabbit	T6391 Invitrogen	Goat		IF- 1:1000 (Figure. 1, S2, S3)
Anti-Rabbit IgG Alexa 594	A21207 Invitrogen	Donkey		IF- 1:4000 (Figure. S1, S6, S7)

Table S2. ZetaView instrument settings for traditional NTA.

Mode	Scatter
Sensitivity	70–80
Shutter	100
Cycles/positions	2/11
Frame rate	30
Maximum Size	1000
Minimum Size	10
Track Length	15
Minimum Brightness	20

Table S3. ZetaView instrument settings for fNTA.

Mode	Scatter	Fl-488/500
Dilution	4000	4000
Sensitivity	75	80
Shutter	100	100

Track length	15	10
Cycle/ positions	2/11	1/11
Low bleach mode	no	yes

Table S4. Western blot quantification data.

MDA-MB-231 Data						
	EV Raw Signal intensity			EV Normalized intensity by experiment		
**CD63**						
Replicate	Vehicle	Eribulin	Paclitaxel	Vehicle	Eribulin	Paclitaxel
1	0.43	0.333	0.425	1.000	0.774	0.988
2	0.565	0.398	0.394	1.000	0.704	0.697
3	1.03	0.852	0.948	1.000	0.827	0.920
4	0.897	0.677	0.737	1.000	0.755	0.822
5	0.471	0.197	0.325	1.000	0.418	0.690
6	0.389	0.284	0.283	1.000	0.730	0.728
**CD9**						
Replicate	Vehicle	Eribulin	Paclitaxel	Vehicle	Eribulin	Paclitaxel
1	0.0187	0.0133	0.0133	1.000	0.711	0.711
2	0.0257	0.0141	0.0137	1.000	0.549	0.533
3	0.0186	0.0136	0.017	1.000	0.731	0.914
4	0.0307	0.021	0.0174	1.000	0.684	0.567
5	0.0164	0.00892	0.0122	1.000	0.544	0.744
6	0.013	0.00703	0.00758	1.000	0.541	0.583
**CD81**						
Replicate	Vehicle	Eribulin	Paclitaxel	Vehicle	Eribulin	Paclitaxel
1	2.74	2.28	2.1	1.000	0.832	0.766
2	3.58	2.58	3.07	1.000	0.721	0.858
3	3.36	3.52	3.01	1.000	1.048	0.896
4	16.9	18	16.1	1.000	1.065	0.953
5	15.9	16.2	13.9	1.000	1.019	0.874
**Flotillin-1**						
Replicate	Vehicle	Eribulin	Paclitaxel	Vehicle	Eribulin	Paclitaxel
1	0.644	0.617	0.541	1.000	0.958	0.840
2	0.608	0.494	0.509	1.000	0.813	0.837
3	0.517	0.497	0.455	1.000	0.961	0.880
4	0.374	0.364	0.325	1.000	0.973	0.869
5	0.851	0.877	0.746	1.000	1.031	0.877

HCC1937 Data

**CD63**						
Replicate	Vehicle	Eribulin	Paclitaxel	Vehicle	Eribulin	Paclitaxel
1	0.39	0.225	0.264	1.000	0.577	0.677
2	1.22	1.01	1.07	1.000	0.828	0.877
3	1.64	1.31	1.41	1.000	0.799	0.860
4	1.48	0.946	1.26	1.000	0.639	0.851
**CD9**						
Replicate	Vehicle	Eribulin	Paclitaxel	Vehicle	Eribulin	Paclitaxel
1	0.105	0.0734	0.0532	1.000	0.699	0.507
2	0.419	0.439	0.404	1.000	1.048	0.964
3	0.489	0.493	0.466	1.000	1.008	0.953
4	0.397	0.239	0.145	1.000	0.602	0.365
**CD81**						
Replicate	Vehicle	Eribulin	Paclitaxel	Vehicle	Eribulin	Paclitaxel
1	3.45	3	3.02	1.000	0.870	0.875
2	0.719	0.605	0.637	1.000	0.841	0.886
3	0.924	0.939	1.1	1.000	1.016	1.190
4	5.5	2.25	3.67	1.000	0.409	0.667
**Flotillin-1**						
Replicate	Vehicle	Eribulin	Paclitaxel	Vehicle	Eribulin	Paclitaxel
1	0.24	0.252	0.249	1.000	1.050	1.038
2	0.612	0.447	0.587	1.000	0.730	0.959
3	0.56	0.625	0.834	1.000	1.116	1.489
4	0.469	0.308	0.39	1.000	0.657	0.832
**ILK**						
Replicate	Vehicle	Eribulin	Paclitaxel	Vehicle	Eribulin	Paclitaxel
1	0.018	0.007	0.012	1.000	0.365	0.646
2	0.016	0.008	0.016	1.000	0.487	1.012
3	0.026	0.010	0.016	1.000	0.366	0.600
**β1-Integrin**						
Replicate	Vehicle	Eribulin	Paclitaxel	Vehicle	Eribulin	Paclitaxel
1	0.309	0.258	0.336	1.000	0.837	1.090
2	0.098	0.072	0.078	1.000	0.736	0.788
3	0.044	0.003	0.006	1.000	0.065	0.137
4	0.206	0.114	0.138	1.000	0.553	0.670
5	0.004	0.003	0.004	1.000	0.778	0.900
